# Skeleton keys in the phage world

**DOI:** 10.1038/s42003-022-04155-5

**Published:** 2022-11-07

**Authors:** Melissa Walker

**Affiliations:** Department of Biology, University of Alabama at Birmingham, Birmingham, AL USA

## Abstract

The viruses infecting bacteria, known as phages, carry a wondrous diversity of enzymes known as endolysins, which are responsible for opening cellular doors, like the membrane or wall, so that newly minted phages are set free. In a recent study, Oechslin and colleagues explored the evolutionary mystery of lactococcal endolysin biodiversity, suggesting that these endolysins are flexible and can be used as kinds of skeleton keys to open a broad range of cellular doors.

Phages naturally infect and kill bacterial cells because they need bacterial internal machinery in order to make new phages, their progeny. A host bacterium is enclosed by a cell membrane, an additional mesh frame made up of peptidoglycan and, in some cases, even a cell wall, all of which a parent phage overcomes using endolysins to ensure new virions can emerge from the host cell.

A recent study^1^ by Oechslin and colleagues looked at the genes that code for endolysins found in phages that infect *Lactococcus*, an important genus of Gram-positive bacteria used in the production of cheese and other dairy products. Endolysins (sometimes with enzymes called holins or spannins) act as keys to open a doorway in the bacterial cell membrane and/or cell wall so that progeny can be released. Once the door is open, the once-corralled phages are able to exit the dying cell in search of their own host bacterium to infect.

The authors obtained 253 lactococcal phage sequences from the NCBI database and classified their respective endolysins according to predicted catalytic domains into 4 groups, named A,B,C and D. They transformed a representative endolysin from each of these groups into *Escherichia coli* cells, isolated the proteins, and characterized their ability to lyse lactococcal host cells in vitro. Three of these four endolysins were capable of lysing cells in solution, while the fourth endolysin needed to be separated from its transmembrane domain before it became active.

**Figure:**
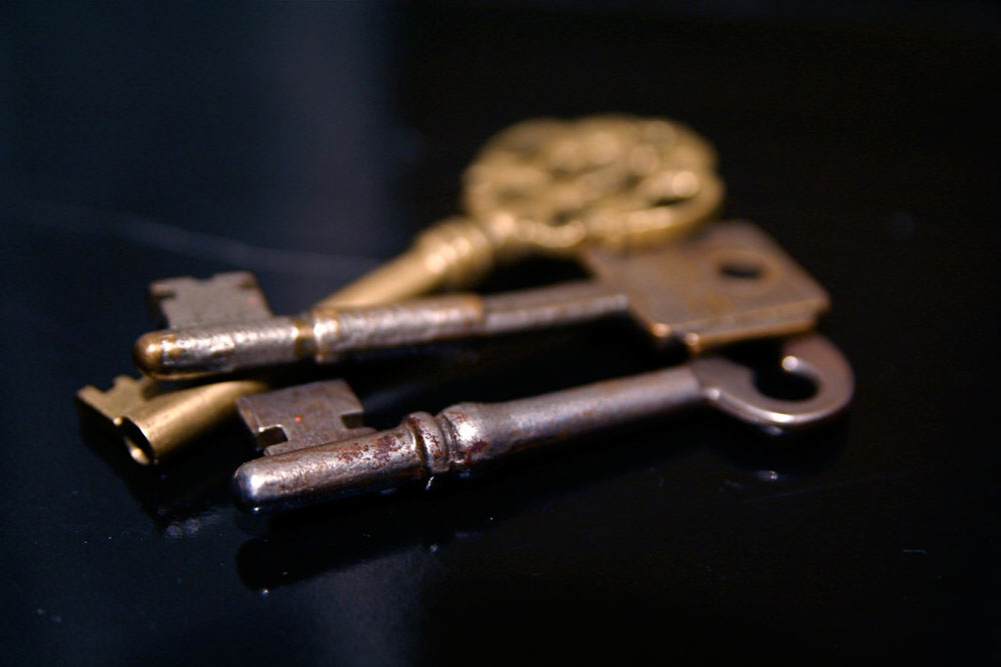
Steven Depolo

Not only were endolysin genes capable of being transformed into *Escherichia coli* cells or even shuttled between closely related phages infecting the same bacterial species, they could also be successfully transferred between unrelated phages infecting different bacterial genera. The recipient phages were, in all cases, able to use the new endolysins to lyse cells and release virions. This discovery demonstrates that endolysins and the genes that produce them are, in a way, like skeleton keys that can unlock multiple types of cellular doors.

The costs of using these endolysin skeleton keys were not synonymous among phages, however. Mutants exhibited an extended latency period, the time during which a phage replicates inside its host, and released a smaller number of progeny viruses, which negatively influenced their competitive advantage in a single host environment. A closer look at this phenomenon, however, revealed that any costs were short-term, since the endolysin genes acquired mutations over time that offset the initial disadvantages.

The implications of this work improve upon our understanding of mechanisms by which phages recombine and equip us with possible defenses translatable to Gram-positive human pathogens. However, many of the multidrug resistant pathogens are Gram-negative organisms. Future work should examine if both the recombination and evolutionary principles established here apply across an expanded genera of Gram-positive bacteria and their phages and additional experiments should also be conducted with Gram-negative bacteria and their phages. These supplementary studies could establish organizing rules of recombination for phages infecting both Gram-negative and Gram-positive bacteria, a tome that has yet to be written.
